# Beyond the shell: exploring polymer–lipid interfaces in core–shell nanofibers to carry hyaluronic acid and β-caryophyllene

**DOI:** 10.3762/bjnano.16.139

**Published:** 2025-11-12

**Authors:** Aline Tavares da Silva Barreto, Francisco Alexandrino-Júnior, Bráulio Soares Arcanjo, Paulo Henrique de Souza Picciani, Kattya Gyselle de Holanda e Silva

**Affiliations:** 1 Laboratório de Sistemas Híbridos, Faculdade de Farmácia, Universidade Federal do Rio de Janeiro (UFRJ), Av. Carlos Chagas Filho, 373 - Cidade Universitária, Rio de Janeiro - RJ, 21941-170, Brasil; 2 Programa de Pós-Graduação em Nanobiossistemas, Universidade Federal do Rio de Janeiro, Duque de Caxias, Brasil; 3 Laboratório de Micro e Nanotecnologia (LMN), CDTS, Fundação Oswaldo Cruz (Fiocruz), Brasil Av., 4036, 107 - Maré, Rio de Janeiro - RJ, 21040-361, Brasil; 4 Divisão de Metrologia e Materiais Instituto Nacional de Metrologia, Qualidade e Tecnologia (INMETRO), Xerém, RJ, Brasil; 5 Laboratório de Biomateriais, Instituto de Macromoléculas Heloisa Mano, Universidade Federal do Rio de Janeiro (UFRJ) – Av. Horácio Macedo, 2030, Rio de Janeiro, Brasil

**Keywords:** co-axial nanofibers, electrospinning, hybrid nanosystem, nanofibers, nanoemulsion, poly(lactic acid)

## Abstract

Hyaluronic acid (HA) and β-caryophyllene (βCp) are two promising agents in biomedical research, each offering unique therapeutic benefits. The successful integration of these compounds into a single, functional nanofiber system presents a significant technical challenge, demanding innovative strategies to ensure their compatibility and sustained activity. This study addresses this critical challenge through the rational design and fabrication of hybrid core–shell nanofibers manufactured via coaxial electrospinning. Poly(lactic acid) (PLA) was used as an outer shell providing structural integrity and effectively encapsulating a core comprising a nanoemulsion containing β-caryophyllene (NE-βCp) alongside HA. A rigorous optimization of the electrospinning process was critical, involving the systematic evaluation of key parameters. This optimization successfully identified the optimal core formulation (1% w/w HA, 2% w/w NE) and process parameters (17 kV applied voltage, 6.25 flow rate ratio (0.04 mL/h inner; 0.25 mL/h outer), 12 cm needle-to-collector distance). These conditions provided highly uniform fibers with an average diameter of 439 ± 100 nm, notably 37% larger than fibers without the lipid core. Furthermore, maintaining ambient relative humidity below 45% proved essential for processing stability. Comprehensive morphological characterization via scanning electron microscopy confirmed the uniformity of the fibers. At the same time, confocal microscopy, cross-sectional imaging, and attenuated total reflectance with Fourier transform infrared (ATR-FTIR) spectroscopy provided compelling evidence for the successful formation of the intended core–shell structure. The resulting nanofibers exhibited surface hydrophobicity, suggesting potential for anti-adhesive membrane applications. Thermal and crystalline analyses demonstrated improved thermal stability upon NE-βCp incorporation. Collectively, these results provide robust evidence for the feasibility of producing multifunctional nanofiber membranes that successfully integrate a polymer–lipid hybrid core encapsulated within a PLA shell, highlighting substantial potential for biomedical applications by overcoming key material integration hurdles.

## Introduction

Driven by the significant potential of biomaterials, recent decades have seen intensive research into novel therapeutic strategies for regenerative medicine [1–4]. Within this scenario, a pivotal current strategy in formulation development focuses on integrating nanocarriers with nanoscale three-dimensional biomaterials, enabling major advancements in the controlled release of diverse bioactive compounds [5–10].

Among the various nanostructured platforms explored for these purposes, nanofibers have gained attention due to their high surface area, adjustable porosity, and robust mechanical properties, which set them apart from conventional fibrous materials. Their flexibility in fabrication allows for integration into a broad range of applications, from drug delivery scaffolds to composite biomaterials, contributing to their increasing relevance in both scientific research and industrial development [11–14].

To leverage the properties of nanofibers and create advanced structures with enhanced functionality, coaxial electrospinning can be used to generate nanofibers allowing for the building of a core–shell structure with desirable properties, taking advantage of the positive characteristics of each component (core and shell materials) [15]. However, achieving high-quality structures for biomedical use with tailored properties requires careful management of various processes, materials, and environmental parameters [16], necessitating thorough optimization of the electrospinning conditions. This includes precise control over process variables, such as high voltage, flow rate, and the distance from the Taylor cone to the collector, which significantly impacts nanofiber morphology [17]. Furthermore, selection of the material to be electrospun is crucial, requiring control over key attributes such as molecular weight, polymer concentration, surface tension, conductivity, and solvent volatility, alongside careful consideration of electrospinning conditions, such as temperature and humidity [18–19].

The polymer selection of nanofibers intended for biomedical applications must prioritize not only mechanical strength, controlled degradation, and moderate hydrophilicity, but also biocompatibility, non-toxicity, and non-carcinogenicity [5].

Adhering to these critical quality requirements for biomedical products, polylactic acid (PLA) was selected for nanofiber production. This biotechnologically derived aliphatic polyester is a rigid thermoplastic known for its biodegradability, biocompatibility, and bioabsorbability [20–21]. Highly attractive due to its versatile physical, chemical, and biological properties, PLA is a suitable option for manufacturing tissue engineering scaffolds, implantable devices, and drug delivery systems, holding recognition as safe and approved for human use by the U.S. Food and Drug Administration (FDA). While PLA exhibits relatively high modulus and strength, it possesses limitations such as low toughness, a slow degradation rate, and high hydrophobicity [18,22–23]. Nevertheless, PLA fibers effectively provide mechanical strength, assist in managing wound exudates, and maintain a moist wound bed. Its properties can also be tailored through blends with different forms of PLA or other biopolymers to achieve desired tensile strength, release profiles, or biodegradation characteristics [24–25]. The growing commercial interest in biopolymers such as PLA is, in part, driven by environmental concerns, climate change, and the depletion of fossil fuel resources, as PLA is derived from renewable sources and is both readily and completely biodegradable.

Hyaluronic acid (HA) is an extensively used component in wound healing applications and naturally occurring in vertebrates [26–27]. It is a key element of the extracellular matrix, providing a gelatinous structure where collagen and elastin fibers are embedded. As an endogenous molecule, it demonstrates ideal biocompatibility and full absorption by human tissues. Its natural origin, high structural conservatism across species, and minimal interaction with blood components make HA and HA-based materials exceptionally biocompatible, a non-negotiable prerequisite for biomedical use. Hyaluronic acid is also recognized and listed in the US FDA inactive ingredient database for various biomedical applications [28–29].

Complementing the regenerative and biocompatible profile of HA, β-caryophyllene (βCp) is another critical component of significant pharmaceutical potential. Among the array of attributes exhibited by βCp, it notably possesses potent analgesic, antioxidant, antimicrobial, and anti-inflammatory effects [30–31]. It is also currently been evaluated as a candidate for skin regeneration due to its effect as a cannabinoid receptor 2 (CB2) ligand, with studies suggesting that CB2 activation by selective agonists can enhance re-epithelialization, reduce pain, and improve inflammatory response during wound healing [32–33]. Chemically, βCp is characterized as a natural bicyclic sesquiterpene found in several plants and essential oils, and as expected for compounds of this class, it exhibits characteristic volatility and low solubility in water [34]. To address these limitations for effective pharmaceutical use, βCp is often formulated within lipid liquid dispersions, such as nanoemulsions, to enhance its stability and improve therapeutic properties [35–38].

While lipid nanosystems offer undeniable advances for the delivery of active compounds, their inherent liquid nature can present formulation challenges, particularly when seeking integration into solid scaffolds like nanofibers. The strategic combination of polymers and lipids in hybrid systems has emerged as a promising approach to overcome these limitations. Incorporating polymer–lipid interfaces within core–shell nanofibers can enhance the system in various scenarios (e.g., improving formulation stability, increasing the encapsulation efficiency, and tailoring the controlled release of therapeutically active molecules [39–40]). This approach has demonstrated promising results, especially for topical drug administration via dressings made of biocompatible polymers containing lipid nanosystems [41–43]. Numerous researchers have successfully encapsulated nanoemulsions into nanofibers for diverse applications, including studies by Kaur et al. (2024) showing superior wound healing with bakuchiol nanoemulsion-loaded electrospun scaffolds compared to that of the gel formulation [44], and Coelho et al. (2021) reporting PVA nanofibers containing chalcone NE as a potential treatment for cutaneous leishmaniasis [45].

Despite these advances in nanofiber composite structures incorporating various nanosystems, a critical gap persists in thoroughly understanding the complex polymer–lipid interactions, particularly in systems designed for the simultaneous co-encapsulation of compounds with vastly different physicochemical properties, such as hydrophilic HA and lipophilic βCp. An in-depth understanding of these specific interactions is essential for precise modulation of encapsulation efficiency, ensuring system stability and achieving tailored kinetic release. Therefore, the primary objective of this study was to thoroughly investigate lipid–polymer interactions within coaxially electrospun PLA nanofibers engineered for hyaluronic acid and βCp co-delivery. The insights gained from this investigation are crucial for bridging this specific knowledge gap and may guide the design of optimized hybrid therapeutic platforms.

## Results and Discussion

**Figure 1:**
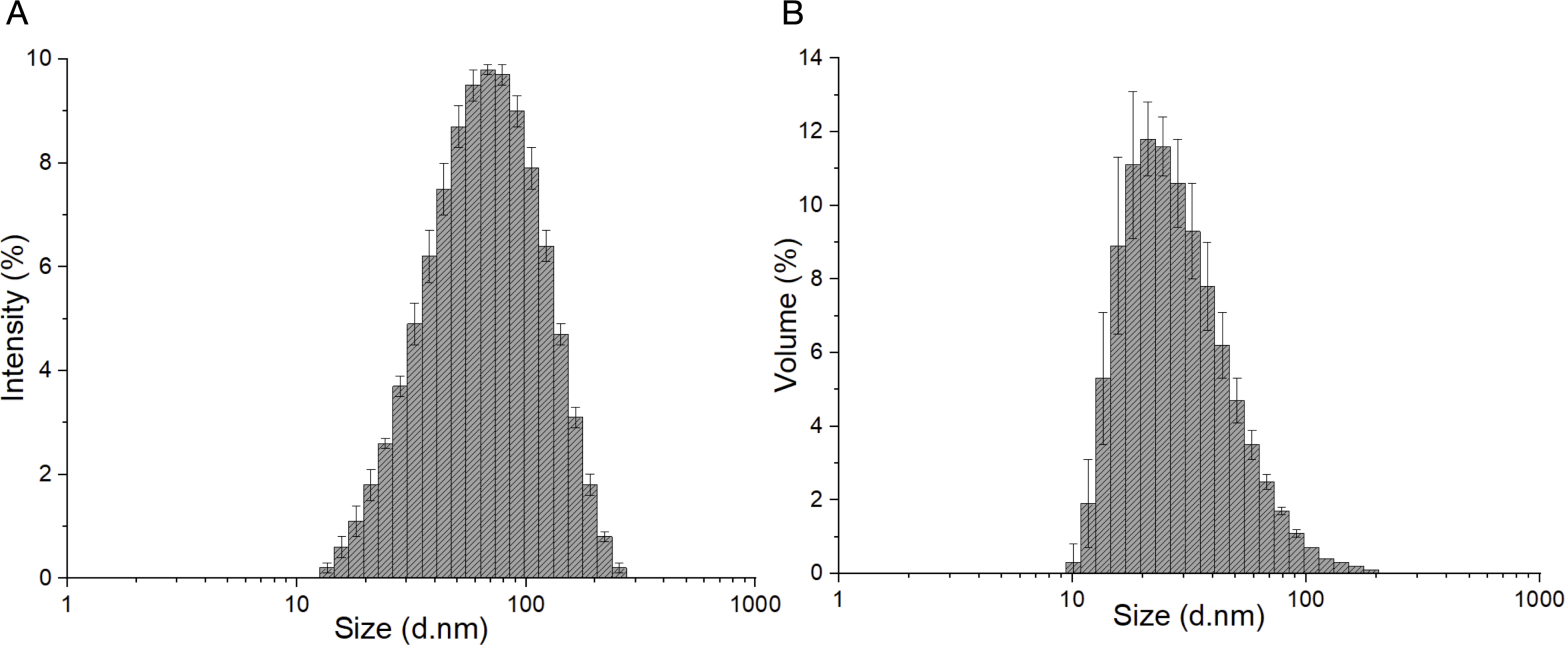
Hydrodynamic diameter distribution of β-caryophyllene nanoemulsion measured by DLS: (A) intensity distribution and (B) volume distribution.

This study aimed to develop and investigate PLA nanofibers impregnated with HA and βCp nanoemulsion. The use of βCp in practice is questioned due to its low aqueous solubility, low viscosity, extreme volatility, and sensitivity to temperature, oxygen, and light [38]. The production of a nanoemulsion was developed as a nanocarrier system designed to enhance both the stability and bioavailability [35] of the compound and enable its incorporation into nanofibers. In this study, a Box–Behnken design was applied to develop an optimized βCp nanoemulsion (NE-βCp). This experimental design employed ultrasonication as the manufacturing method with the oil: βCp ratio (0–80%), surfactant concentration (2–20%), and HLB (10–15) as the independent variables (data not shown). Based on the preliminary results, a sample was selected for this study. It can be observed that formulations exhibited droplet mean sizes in the nanoscale range, varying from 58 nm. The formulations showed a unimodal distribution with a polydispersity index (PDI) range of 0.218. Most of the formed nanoemulsions are small in size and well distributed (Figure 1).

The most favorable processing conditions for obtaining uniform fibers – free of surface roughness and with fewer beads – were determined based on morphological analysis. Varying the process parameters and the concentration of the constituent(s) seems to be an effective way to control the morphology of the electrospun mats, especially the diameter of the fibers, as demonstrated by Coelho et al. [45]. This study investigated the influence of core composition on fiber formation by analyzing the concentrations of the core components and the process flow rate as variables.

**Figure 2:**
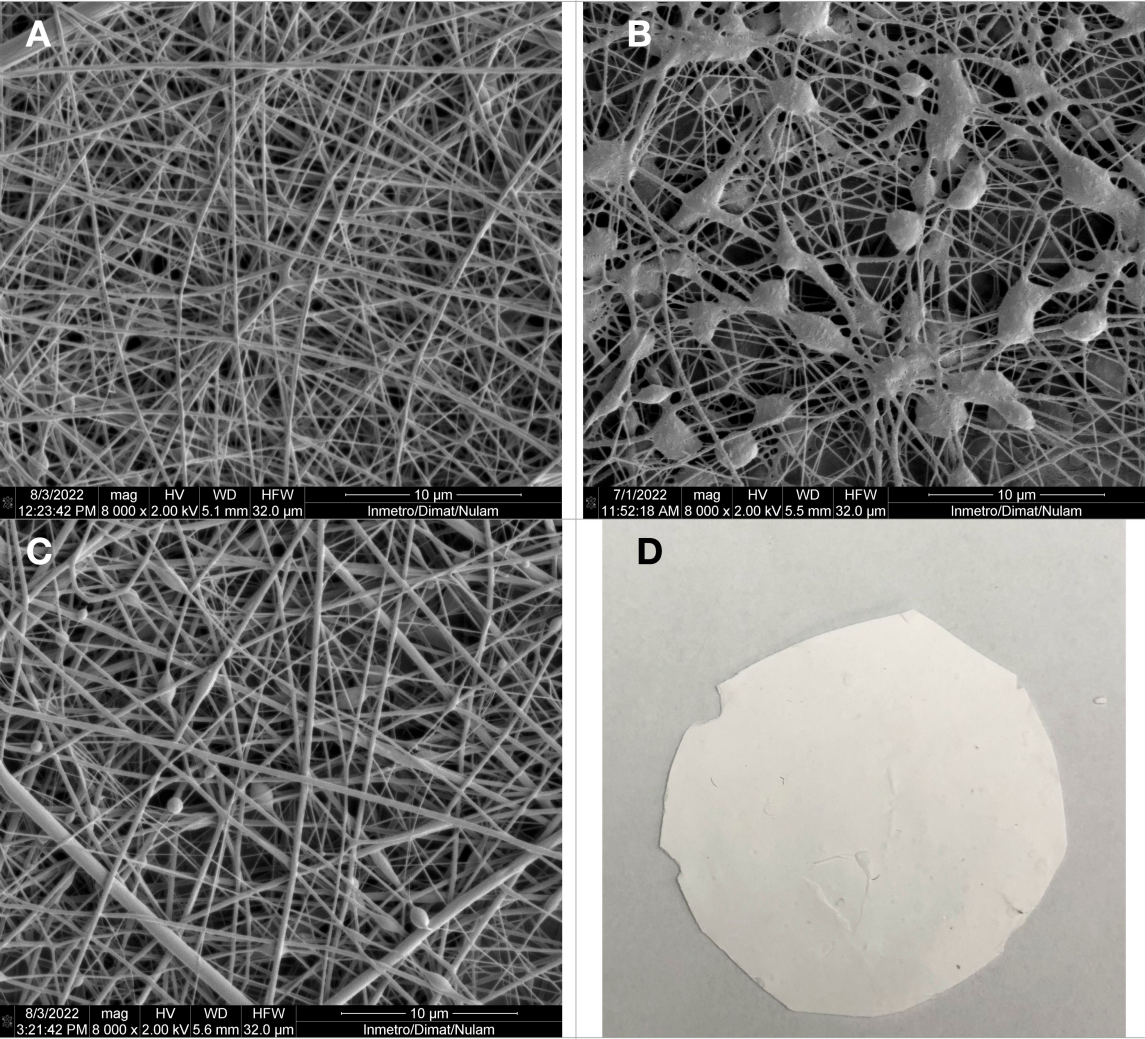
SEM images of nanofibers obtained from the composition: (A) PLA 20%_(w/w)_ and HA 1%_(w/w)_; (B) PLA 20%_(w/w)_, HA 1%_(w/w)_, and NE 5%_(w/w)_; (C) PLA 20%_(w/w)_, HA 1%_(w/w)_, and NE 2%_(w/w)_; and (D) picture of a nanofiber mat. The scale bars are 10 μm and the magnification is 8000×.

It was observed that the concentration of NE-βCp in the core had a significant impact on fiber morphology, as illustrated in Figure 2. The presence of NE-βCp led to an increase in the number of beads and the formation of fibers with a more dispersed structure, likely reflecting insufficient polymer chain entanglement and Taylor cone instability. This behavior appears to result from interactions between NE-βCp droplets and HA chains, which may disrupt the jet and hinder solvent evaporation within the core, as also reported by Ricaurte et al. (2022) [46]. Air relative humidity was additionally found to influence solvent evaporation and the formation of uniform HA fibers. Similar to the observations reported by Yao et al. (2013), a stable process without interruptions was only achieved when the relative humidity was below 45% [47].

**Figure 3:**
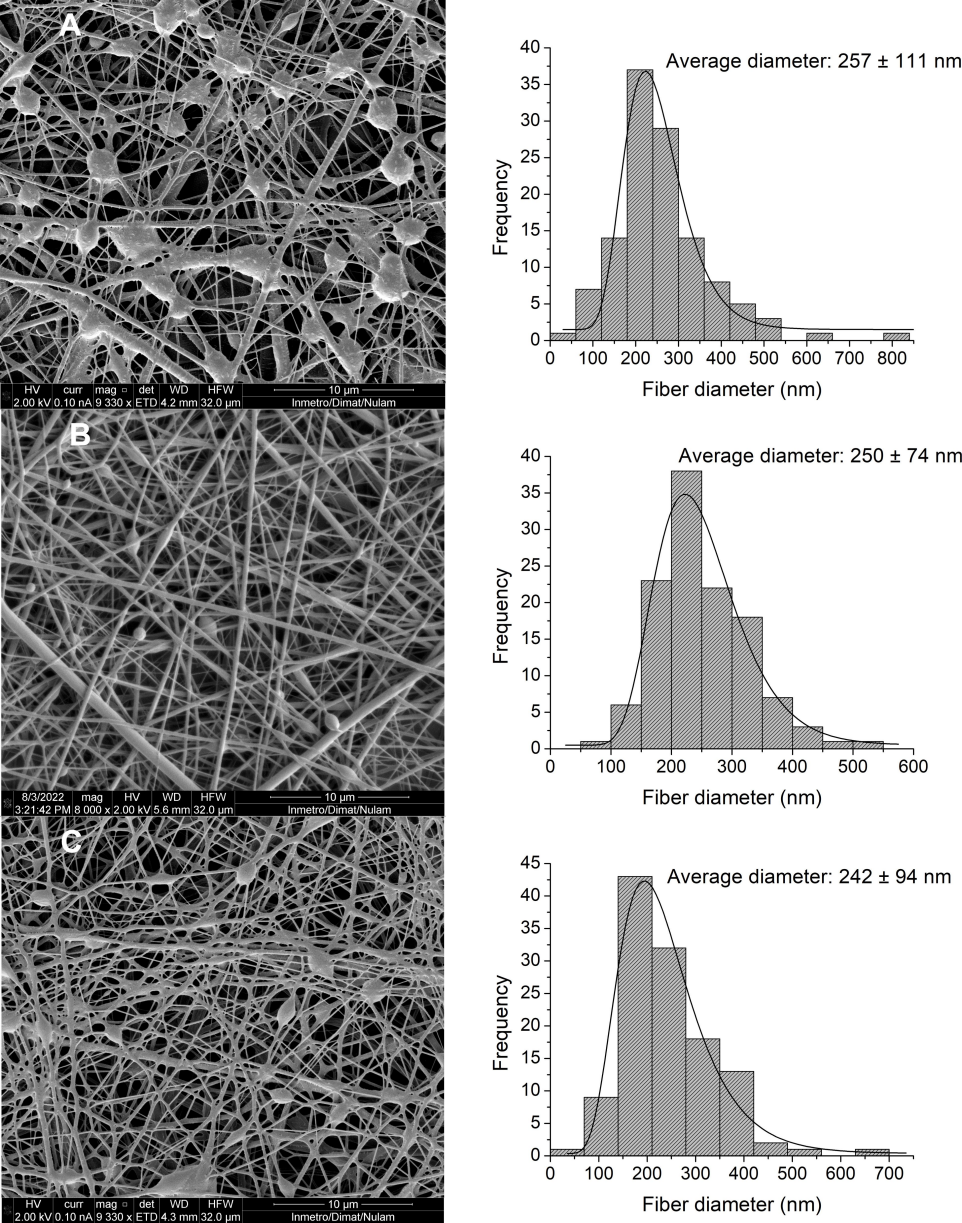
SEM images and histograms with diameter frequencies of nanofibers obtained from the composition: NE 2%_(w/w)_, PLA 20%_(w/w)_, and (A) HA 0.75%_(w/w)_; (B) HA 1.00%_(w/w)_; and (C) HA 1.25%_(w/w)_. The scale bars are 10 μm. In picture (B), the magnification is different (8000×).

Regarding the NE-βCp concentration, it was evident that values above 2% (Figure 2B) led to the formation of more heterogeneous fibers with a higher density of beads, likely because the increased droplet content hindered proper flow and elongation of the polymer jet. Based on these preliminary results, the NE concentration and flow rates (outer flow rate: 0.25 mL/h; inner flow rate: 0.04 mL/h) were fixed in subsequent experiments to investigate the influence of HA concentration on nanofiber formation. Given that HA is a high-viscosity polymer [48], its concentration in the core significantly affects the overall viscosity of the solution, potentially causing instability in the Taylor cone and leading to bead formation and other morphological defects in the fibers [49]. Micrographs revealed the morphological changes resulting from different HA concentrations. Figure 3 shows that no significant variations in the average fiber diameter were observed with increasing HA concentrations: diameters of 257 ± 111 nm, 250 ± 74 nm, and 242 ± 94 nm were obtained for HA concentrations of 0.75%, 1.00%, and 1.25%, respectively. However, the micrograph corresponding to 0.75% HA showed a higher density of beads, suggesting insufficient polymer chain entanglement, which may have impaired fiber stretching during the electrospinning process. Among the tested concentrations, 1.00% HA yielded the most uniform fibers. This effect may result from a more favorable balance between chain entanglement and viscosity: compared to 0.75%, the solution exhibited increased entanglement, while its viscosity remained lower than that of the 1.25% solution, facilitating better core dragging by the shell solution.

In the coaxial electrospinning process, the optimal ratio between the shell and core flow rates is crucial to ensure proper incorporation of the core material. When this ratio is excessively high, the core flow becomes insufficient relative to the shell flow, resulting in fibers with a discontinuous or poorly defined core [50]. Conversely, when the core flow is too high, structural instability and bead formation may occur, as the shell solution may not efficiently envelop or drag the core solution [51–52]. In this study, a flow rate ratio of 12.5 (outer flow rate: 0.50 mL/h; inner flow rate: 0.04 mL/h) was associated with the formation of thinner fibers and numerous beads, likely due to limited core incorporation and the generation of fine PLA nanofibers with bead-like structures containing core material. Upon reducing the ratio, the resulting fibers were thicker and exhibited fewer beads, suggesting improved core incorporation. Therefore, for sample preparation, a flow rate ratio of 6.25 was maintained, with an outer flow rate of 0.25 mL/h and an inner flow rate of 0.04 mL/h, as this ratio prevented severe dripping and ensured a more stable Taylor cone. Having characterized the influence of core composition on fiber formation, the next step involved evaluating the effects of electric field intensity, which is responsible for polymer chain stretching and the speed of fiber deposition, on fiber morphology. Specifically, the impact of varying the needle-to-collector distance (12 and 15 cm) and the applied voltage (15, 17, and 19 kV) was assessed. The important physicochemical properties of these structures are highly related to conformational changes in the polymeric chain caused by the spinning process [49,53].

**Figure 4:**
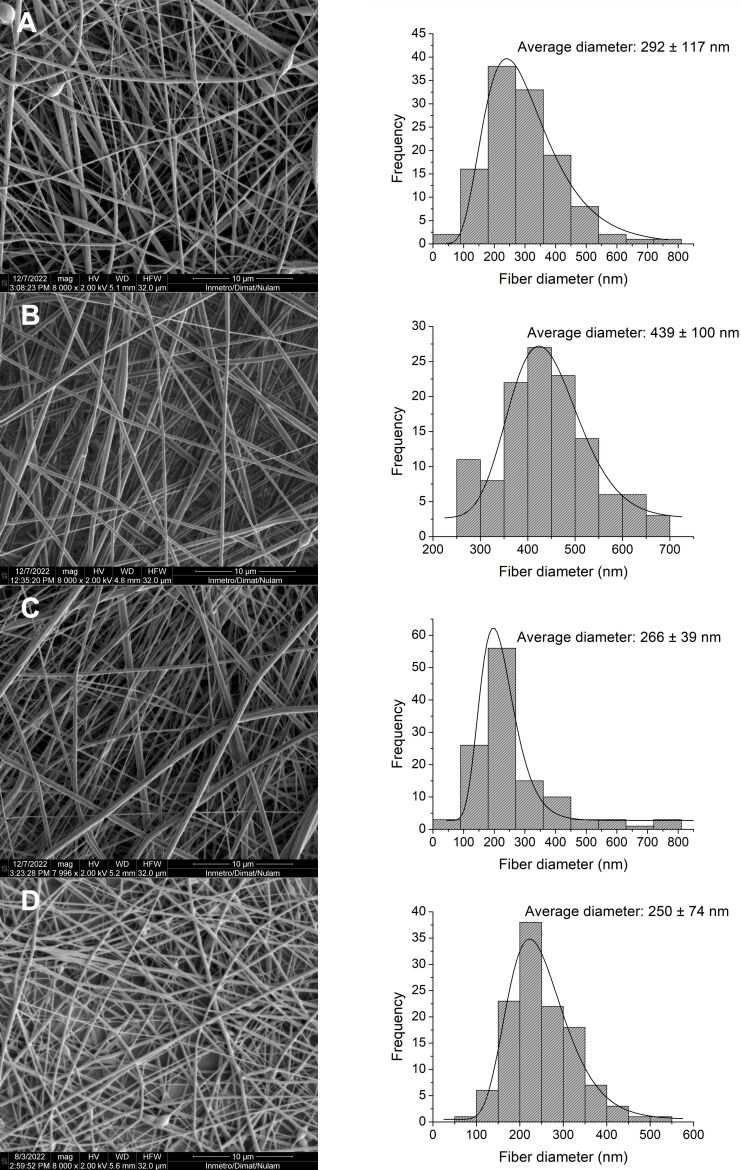
SEM images and histograms with diameter frequencies of nanofibers obtained from the composition: HA 1%_(w/w)_, NE 2%_(w/w)_, and PLA 20%_(w/w)_ electrospun at (A) 12 cm and 19 kV, (B) 12 cm and 17 kV, (C) 12 cm and 15 kV, and (D) 15 cm and 17 kV. The scale bars are 10 μm, and the magnification is 8000×.

Initially, the influence of the travel distance of the polymer jet on fiber formation was examined (Figure 4). This critical parameter exerts a notable influence on the characteristics of the resultant nanofibers. A reduction in the needle-to-collector distance from 15 (Figure 4D) to 12 cm (Figure 4B) resulted in a significant increase in the average fiber diameter, from 250 ± 74 to 439 ± 100 nm. This phenomenon is likely associated with the enhancement of the electric field strength at shorter distances, which accelerates fiber deposition and reduces the time available for jet elongation, ultimately leading to the formation of thicker fibers. Additionally, a stronger electric field may reduce Rayleigh instability and stabilize the Taylor cone [49]. Although the diameter distribution was narrower at 15 cm, the 12 cm distance was selected for subsequent experiments due to the enhanced Taylor cone stability and reduced bead formation. The influence of the distance between the needle and the collector is dependent on the polymer system used. For this study, we determined the optimal distance required to maximize the flight time for solvent evaporation, without compromising the applied electric field, to favor the formation of fibers with adequate morphology and uniformity.

With a fixed distance of 12 cm between the needle and the collector, the influence of voltage variation on fiber morphology was then investigated. Similar to other process parameters, the critical voltage varies among different polymer systems, and optimizing this value is crucial for achieving optimal morphology in the resulting fibers [54]. Increasing the voltage from 17 (Figure 4B) to 19 kV (Figure 4A) resulted in the formation of thinner fibers (292 ± 117 nm), probably due to the amplified repulsive forces which consequently induced stretching. However, it was also observed that more beads and a broader diameter distribution were present, suggesting that the critical voltage threshold may have been exceeded, resulting in the formation of two separate jets: one from the core and another from the shell, ultimately producing monolithic fibers. Reducing the voltage to 15 kV (Figure 4C) yielded fibers with an average diameter of 266 ± 139 nm, no observable beads, and a heterogeneous diameter distribution.

### Structural characterization of nanofibers

Core–shell nanofibers create a protective environment for bioactive agents within the core, preserving their activity while enabling controlled release. By tailoring the shell architecture, the release profile can be modulated, and the initial burst release minimized [7]. Beyond drug delivery, an ideal biomedical scaffold should support cellular attachment while providing effective drug release; balancing these functions is crucial for promoting tissue formation. Achieving this balance in electrospun scaffolds remains challenging, as modifications that improve release kinetics or protect the core can sometimes compromise biocompatibility or cellular interactions [55].

**Figure 5:**
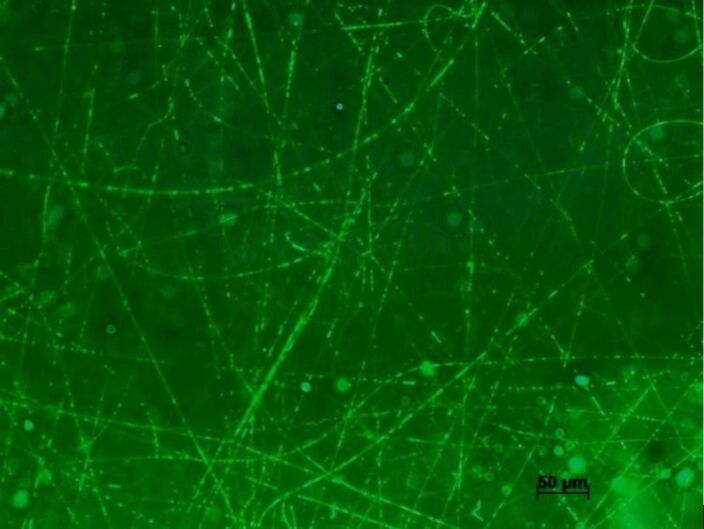
Confocal microscopy images of nanofibers containing fluorescein. The scale bar is 50 μm.

To evaluate the structure of the nanofibers produced, confocal microscopy was used under a fluorescent filter to study the morphologies of scaffold types throughout their thickness and assess the core structure of HA+NE2/PLA nanofibers. Fluorescein, a fluorescent dye, was added to the HA+NE mixture to facilitate its encapsulation within the core during the electrospinning process. As shown in Figure 5, the presence of fluorescein was confirmed by its fluorescence emission under incident light, indicating that the electrospun fibers predominantly displayed continuous cores. This suggests that the established coaxial electrospinning parameters successfully supported the formation of nanofibers with HA+NE cores. The limited discontinuities identified in the fiber structure were attributed to instabilities in the electrospinning process, likely resulting from the low core flow rate (0.04 mL/h). Despite these minor defects, the majority of the fibers exhibited continuous and well-defined cores, confirming the consistency of the process and their potential suitability for subsequent applications. The observed discontinuities could act as preferential sites for accelerated diffusion and burst release of encapsulated compounds, potentially compromising a sustained release profile while being advantageous for applications requiring an initial burst. To improve fiber homogeneity, strategies such as fine-tuning the core–shell flow rate ratio and optimizing solution viscosities can be employed, enhancing coaxial jet stability and promoting more continuous cores [16].

The evaluation of the cross-section before and after core removal is a key technique for assessing the core–shell structure of coaxial fibers. This method provides insight into the structural changes resulting from the release of hydrophilic core content and potential pore formation on the shell surface. For this study, the HA+NE2/PLA sample was analyzed before and after core removal.

**Figure 6:**
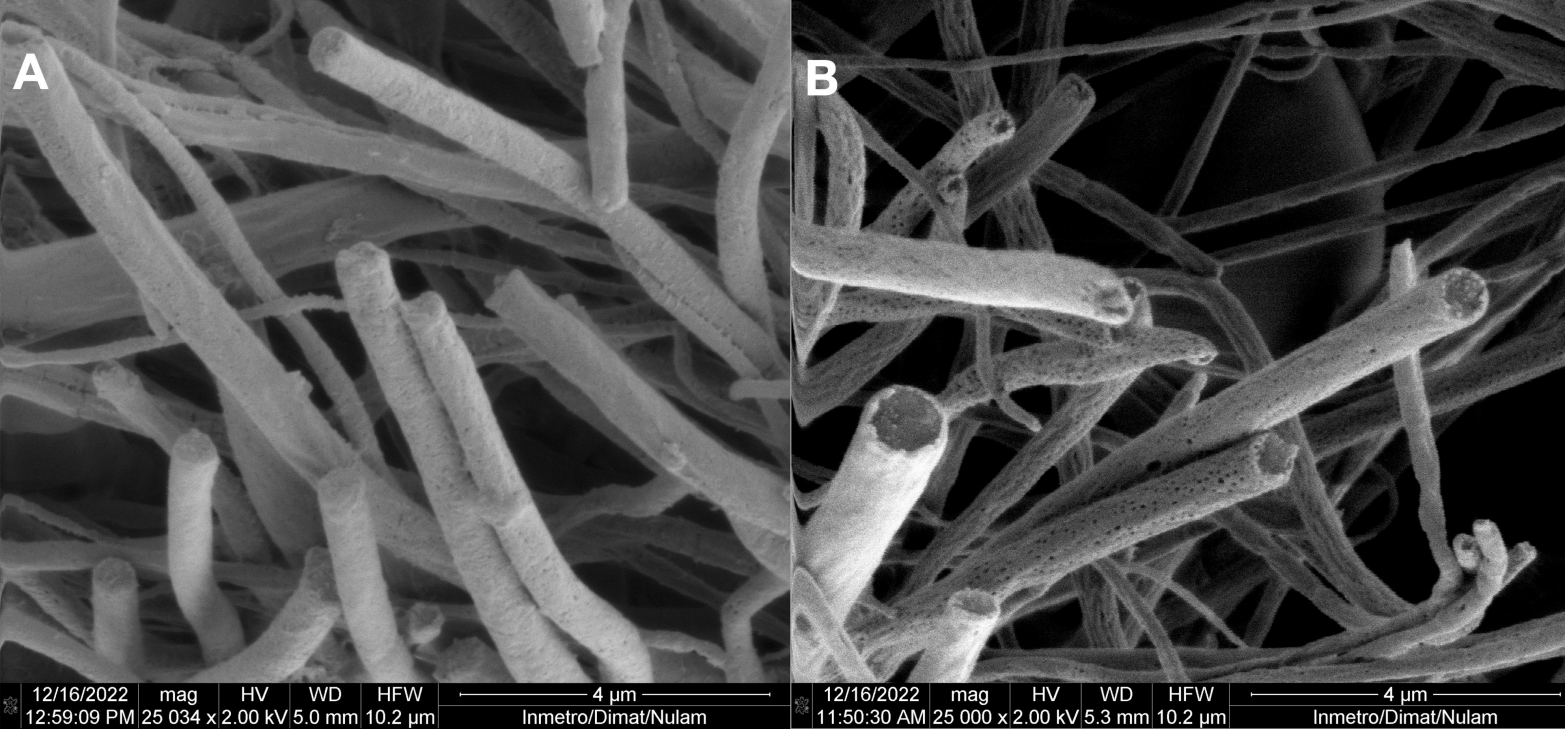
SEM images of the cross-section of nanofibers obtained before (A) and after (B) core removal. The scale bars are 4 μm, and the magnification is 25000×.

Before core removal, the fibers exhibited a filled core structure, as depicted in Figure 6A. Sequential washing with water and ethanol, followed by drying and cryofracture, effectively solubilized and removed the core material, yielding hollow fiber structures (Figure 6B). These observations confirmed the successful formation of a core–shell structure via coaxial electrospinning, using the optimized parameters from the morphology study. Additionally, Figure 6B highlights the presence of pores on the PLA shell surface after washing, suggesting that core removal occurred through diffusion facilitated by these pores. The pore formation on the shell surface is a potential mechanism for regulating the release of the core content [56–57]. A biomedical scaffold must possess suitable pore size and a high level of porosity. The porosity of the scaffold in tissue engineering applications should be adequate. A study by Nguyen et al. (2012) compared the release profiles of salicylic acid encapsulated within cores protected by porous and nonporous PLA shells. Over a period of five days, the release from fibers with porous shells was approximately five times greater than that from nonporous counterparts, likely due to increased water ingress through the pores, facilitating access to the core [58]. This possibility is also described by Wang and Xu (2018), who successfully prepared tea polyphenol-loaded porous core–shell fibers by controlling coaxial electrospinning parameters [56].

ATR-FTIR spectroscopy has been widely used to evaluate the integrity of core–shell structures and confirm the presence of the core material within electrospun fibers. A well-formed core–shell structure is indicated by FTIR spectra showing the absence of characteristic core bands and the exclusive presence of shell bands [59–61]. As reported by da Silva et al. (2019), inadequate flow rates during the production of coaxial nanofibers can lead to incomplete core incorporation, resulting in monolithic fibers that are identifiable by their spectral signatures [61]. To evaluate the surface composition of nanofiber membranes, ATR-FTIR analysis was performed on PLA (monolithic), HA/PLA, and HA+NE2/PLA nanofiber samples, along with pure HA powder.

**Figure 7:**
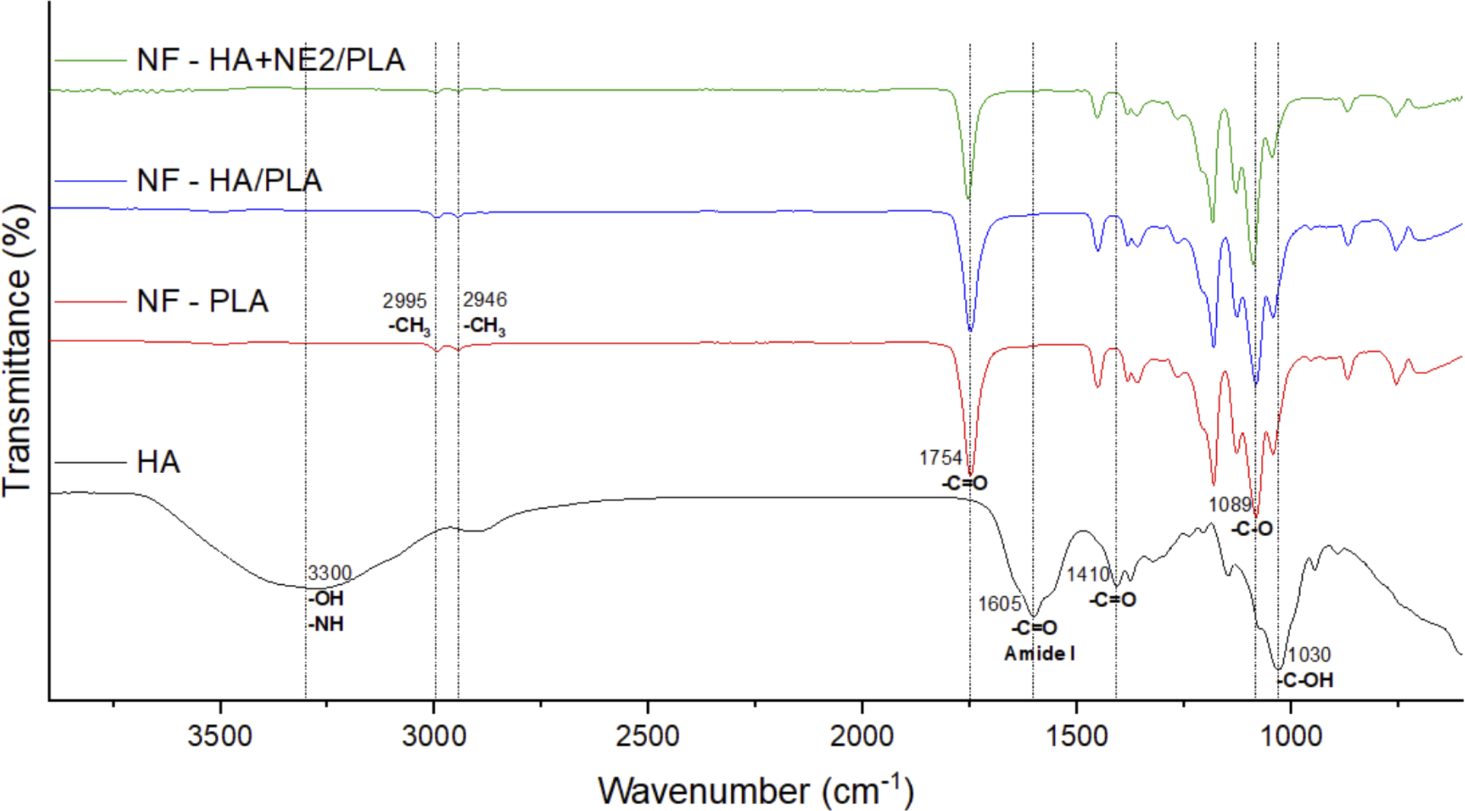
FTIR spectra of bulk HA (black), PLA nanofiber (red), nanofiber composed of HA 1%_(w/w)_ and PLA 20%_(w/w)_ (blue), and nanofiber composed of HA 1%_(w/w)_, NE 2%_(w/w)_, and PLA 20%_(w/w)_ (green).

The obtained spectra (Figure 7) confirmed effective shell coating, as only the characteristic bands of PLA were detected in the core–shell nanofibers. All three nanofiber membranes (NF-PLA, NF-HA/PLA, and NF-HA+NE2/PLA) exhibited PLA-specific bands at 2995, 2946, 1759, and 1089 cm^−1^, corresponding to the asymmetric stretching vibration of –CH_3_, symmetric stretching of –CH_3_, and stretching vibrations of C=O and C–O, respectively [62]. The HA powder spectrum displayed bands at 3300, 1605, 1410, and 1030 cm^−1^, which were attributed to the overlapping O–H and N–H stretching of hydroxyl and amide groups, the amide I C=O stretching, the symmetric stretching of C=O, and the C–O stretching, respectively [63–64]. In comparison, the absence of HA-specific bands at 3300 and 1605 cm^−1^ in the nanofiber spectra confirmed the complete encapsulation of the core material by the PLA shell. This observation aligns with the cross-sectional analysis results, where a coated core–shell structure was evident following the removal of the HA+NE2 core.

### Thermal behavior and crystallinity of nanofibers

**Figure 8:**
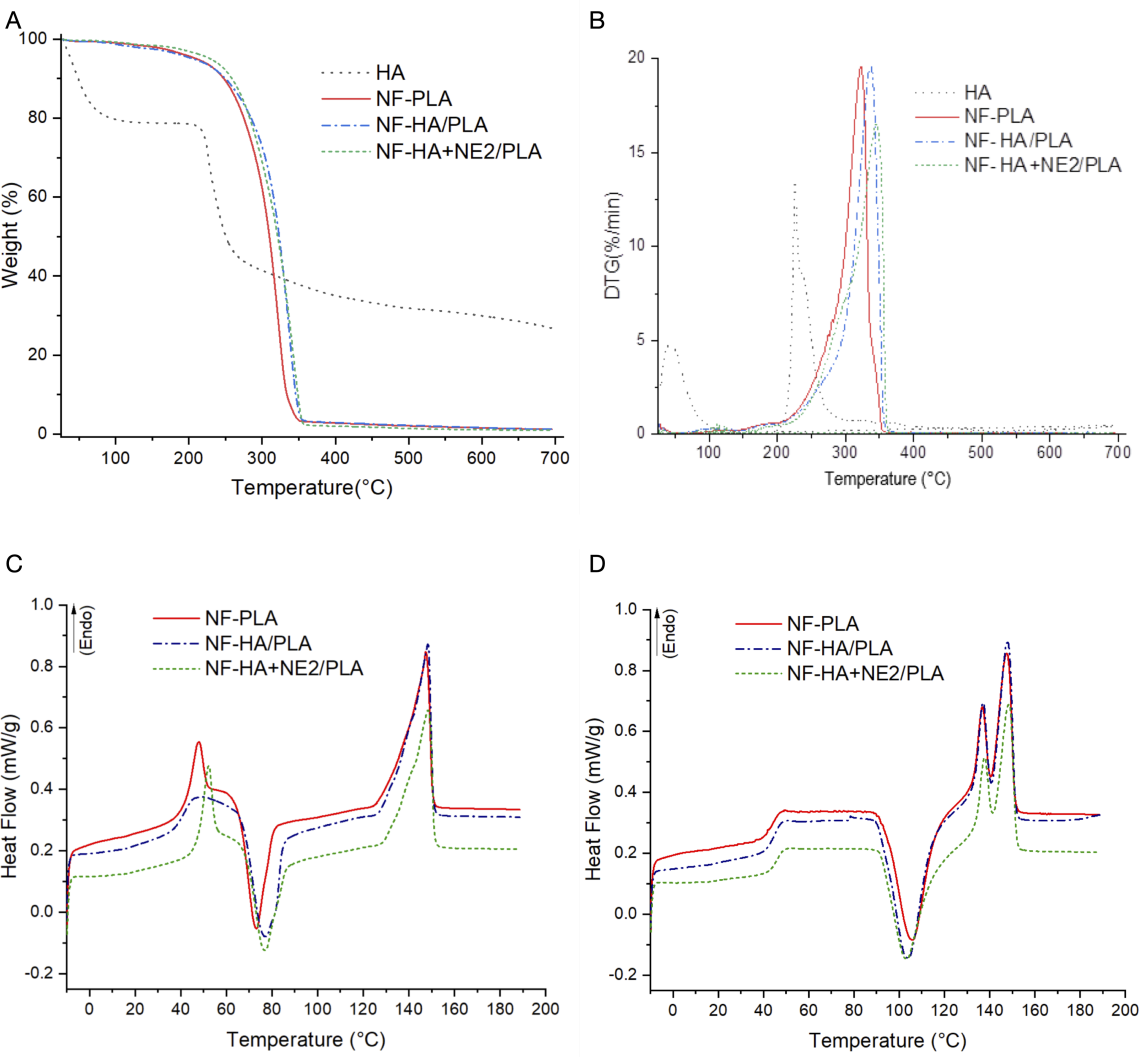
Thermal characterization plots of neat HA, PLA, and nanofiber compositions. Thermogravimetric analysis (A), DTG curves (B), and DSC thermograms: (C) first heating and (D) second heating.

The thermal properties and crystallinity of NF-PLA (monolithic), NF-HA/PLA, and NF-HA+NE2/PLA nanofibers were analyzed using thermogravimetric analysis (TGA), X-ray diffraction (XRD), and differential scanning calorimetry (DSC). Thermal behavior and crystallinity of the nanofibers mats of βCp nanoemulsion-loaded PLA are displayed in Figure 8. The TGA and DTG curves provide insights into the thermal stability and decomposition behavior of the nanofibers [65]. At the same time, the DSC and XRD analyses complement the study by elucidating the crystallinity and thermal transitions of the samples. Figure 8A and Figure 8B illustrate the mass variation curves as a function of temperature, along with the first derivative of mass variation concerning temperature (DTG), obtained from TGA. In the TGA curve of the HA bulk, a degradation profile with three main stages of mass loss was observed, as previously described by Ahire et al. [66]. The first stage of mass loss occurred at temperatures up to 220 °C. In this stage, approximately 21% of the mass was lost due to the dehydration of HA chains, resulting from the evaporation of water molecules. HA polymer chains are characterized by a high capacity for water adsorption and retention, owing to their large number of pendant hydrophilic groups and atoms capable of forming hydrogen bonds, such as nitrogen and oxygen. The second and third stages corresponded to the degradation and cleavage of the HA chains, resulting in mass losses of approximately 40% between 220 and 300 °C, and 13% between 300 and 700 °C [66–67]. Therefore, about 26% of the HA remained thermally stable up to 700 °C, yielding a residual mass that did not decompose at the analyzed temperature.

Regarding the nanofibers, the TGA and DTG curves indicated a single decomposition stage associated with the cleavage of polymer chains. No significant differences were observed in the *T*_onset_ and *T*_max_ temperatures among the different nanofiber groups, likely due to the high PLA content (≈99%). Monolithic PLA nanofibers exhibited a *T*_onset_ of 286.8 °C and a *T*_max_ of 322.5 °C, values consistent with those reported by Vidal et al. [68]. The incorporation of an AH core into the PLA nanofibers led to an increase in *T*_onset_ to 301.2 C and *T*_max_ to 337.4 °C. Similarly, the addition of the HA+NE core also increased the *T*_onset_ to 294.7 °C and the *T*_max_ to 343.6 °C. These results indicate that the presence of the core did not reduce the thermal stability of the nanofibers, suggesting that the hybrid HA+NE core may have enhanced this stability, likely due to an interaction between the core and the shell.

Complementing the thermal analysis performed by TGA, DSC was used to obtain detailed information on the thermal and crystallinity properties of the nanofibers through the values of *T*_g_, *T*_cc_, *T*_m_, Δ*H*_m_, Δ*H*_cc_, and *X*_c_%. The DSC analysis provided heating curves and thermal transitions for the first and second heating cycles of the nanofibers. The first heating cycle offers insights into the polymers after processing by electrospinning, while the second heating cycle reveals characteristics of the material before processing. The values for each thermal event are presented in Table 1 (first heating) and Table 2 (second heating). All fibers exhibited a typical semicrystalline polymer profile, featuring an endothermic peak corresponding to the melting of crystallites, an exothermic peak related to the crystallization of polymer chains into spherulites, and a baseline shift indicative of the glass transition of the amorphous regions. Due to the high PLA content in the fibers, all three analyzed samples exhibited thermal behavior similar to that of monolithic PLA nanofibers.

**Table 1:** DSC data corresponding to the first heating.^a^

Sample	*T*_g_ (°C)	*T*_cc_ (°C)	Δ*H*_cc_ (J/g)	*T*_m1_ (°C)	*T*_m2_ (°C)	Δ*H*_m_ (J/g)

NF-PLA	43.7	105.9	37.9	136.6	147.5	33.5
NF-HA/PLA	44.3	104.2	42.7	137.4	148.0	35.7
NF-HA+NE2/PLA	45.6	103.4	33.0	137.8	148.5	29.6

^a^Glass transition temperature (*T*_g_), crystallization temperature upon cooling (*T*_cc_), melting temperature of the α crystalline form (*T*_m1_,), melting temperature of the β crystalline form (*T*_m2_), melting enthalpy (Δ*H*_m_), and crystallization enthalpy (Δ*H*_cc_).

**Table 2:** DSC data corresponding to the second heating.^a^

Sample	*T*_cc_ (°C)	Δ*H*_cc_ (J/g)	*T*_m_ (°C)	Δ*H*_m_ (J/g)	*X*_c_% (PLA)

NF-PLA	72.8	23.8	147.3	35.4	12.4
NF-HA/PLA	76.2	30.5	148.3	38.3	8.4
NF-HA+NE2/PLA	76.3	22.0	148.4	31.0	9.8

^a^Crystallization temperature upon cooling (*T*_cc_), melting temperature (*T*_m_), melting enthalpy (Δ*H*_m_), crystallization enthalpy (Δ*H*_cc_), and degree of crystallinity (*X*_c_%).

In the first heating cycle (Figure 8C), all three samples displayed an exothermic peak between 72–77 °C, corresponding to the cold crystallization temperature (*T*_cc_), which indicates the crystallization of chains that were not fully crystallized during the electrospinning process. Additionally, an endothermic peak at approximately 148 °C was observed in all samples, characteristic of the PLA melting temperature (*T*_m_). The appearance of two melting peaks between 135–150 °C during the second heating cycle (Figure 8D) is associated with two distinct crystalline forms of PLA, the α-form and a metastable β-form. The latter may have formed through recrystallization and reordering of α-crystals upon remelting [69–70]. In the second heating cycle (Figure 8D), a baseline shift was observed between 43–45 °C, corresponding to the glass transition temperature (*T*_g_) of PLA and related to enthalpic relaxations in the amorphous regions.

**Figure 9:**
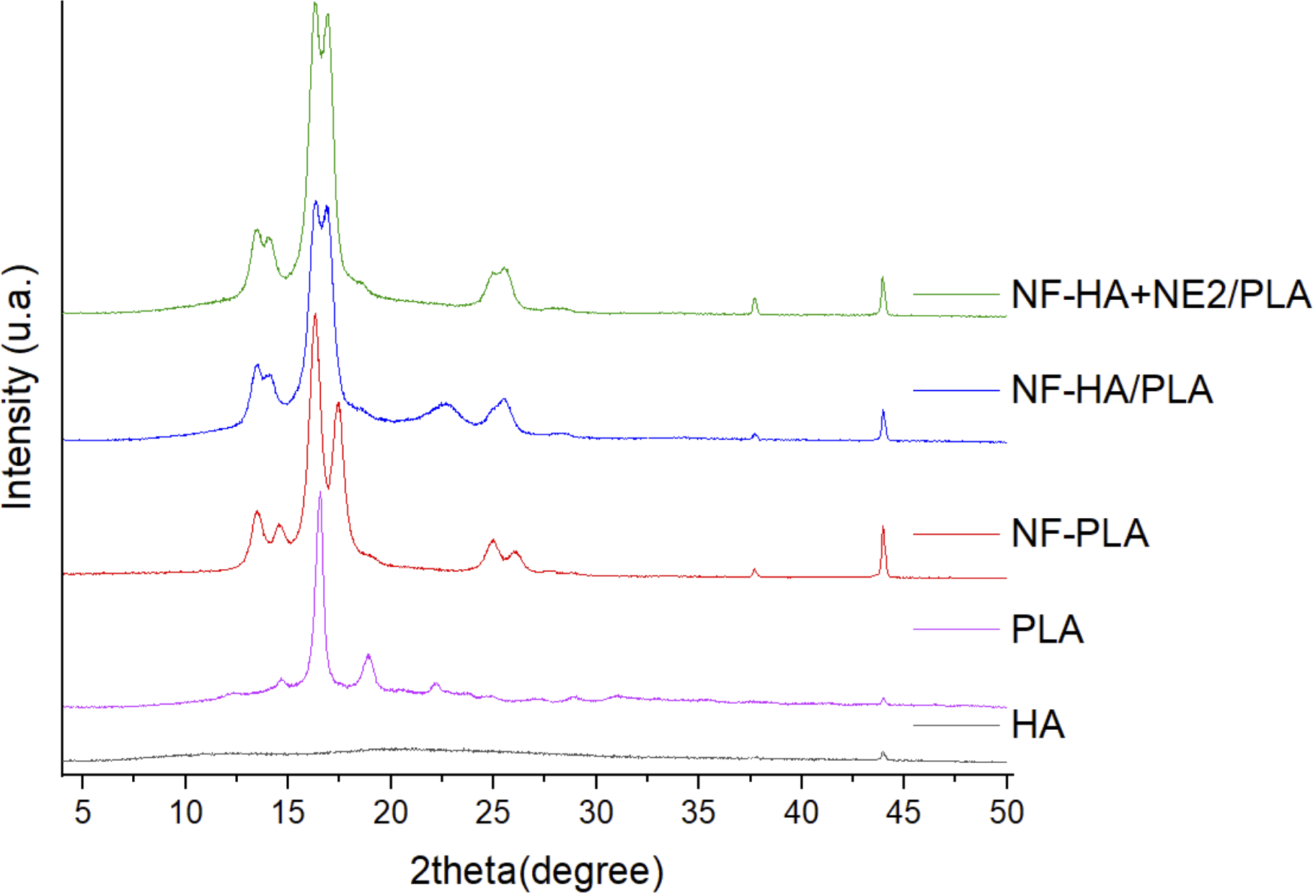
X-ray diffractograms of bulk HA (black), bulk PLA (rose), PLA nanofiber (red), nanofiber composed of HA 1%_(w/w)_ and PLA 20%_(w/w)_ (blue), and nanofiber composed of HA 1%_(w/w)_, NE 2%_(w/w)_, and PLA 20%_(w/w)_ (green).

X-ray diffraction (XRD) analysis was also performed to compare the crystallinity and characteristic peaks of the produced nanofibers. Figure 9 presents the diffractograms of the NF-PLA, NF-HA/PLA, and NF-HA+NE2/PLA nanofibers, as well as the HA and PLA powders for comparison purposes. As expected, the HA powder exhibited the typical profile of an amorphous polymer, with a broad amorphous halo between 2θ = 15° and 30°. The PLA powder displayed a characteristic semicrystalline PLA diffraction pattern, with four prominent peaks at 2θ = 14.7°, 16.6°, 18.9°, and 22.2° [22,71], along with lower-intensity peaks at 2θ = 24.9°, 27.3°, 29.0°, and 31.0°. In the NF-PLA diffractogram, three additional peaks were observed at 2θ = 13.5°, 26.1°, and 37.7°, as well as an increase in intensity and a shift of the 2θ = 18.9° peak to 2θ = 17.5°, along with the disappearance of the 2θ = 22.2° peak. The core–shell nanofiber samples exhibited very similar diffraction patterns, indicating that the polymers adopted the same structural characteristics during processing. The common peaks between the two coaxial fiber conditions were observed at 2θ = 13.5°, 14.1°, 16.3°, 16.9°, 25.5°, 37.7°, and 43.9°, all of which were also present in the NF-PLA pattern. Additionally, the NF-HA/PLA sample exhibited a peak at 2θ = 22.6°, which is also present in the PLA powder pattern.

No crystalline phases of HA or NE were detected. The degree of crystallinity of the nanofibers and PLA, calculated from the crystalline fraction area obtained by integration of the diffraction peaks, is presented in Table 3 using the OriginLab software. The crystalline fraction refers to the portion of the material that exhibits crystallinity relative to the total material. It can be determined by measuring the area of the diffraction peaks corresponding to the crystalline structure, excluding the area attributed to the amorphous portion [72]. The PLA powder exhibited a crystallinity degree of 27%, whereas the PLA nanofibers showed a crystallinity degree of 53%. This increase in crystallinity after the electrospinning process can be attributed to the stretching of polymer chains, resulting in greater molecular organization [69].

**Table 3:** Degree of crystallinity obtained by XRD.

Sample	*X*_c_%

PLA	27
NF-PLA	53
NF-HA/PLA	44
NF-HA+NE2/PLA	52

As also observed in the DSC analysis, the XRD patterns revealed lower crystallinity for the nanofibers containing an HA core compared to the monolithic PLA fibers. The prominent peaks, centered at approximately 2θ = 13°, 16°, and 19°, appeared broadened and merged when the amorphous HA core was incorporated. This reduction in crystallinity, resulting from an increase in amorphous regions within the material, may be attributed to the presence of an amorphous core and/or a decrease in the available space for PLA molecular organization within the shell structure.

The crystallinity of the PLA shell can be correlated with its hydrolytic degradation behavior and, consequently, with its ability to control the release of the active compound incorporated in the core. These findings indicate that the crystallinity of the material can be modulated to tailor the degradation rate for specific applications. The semicrystalline nature of PLA results in the formation of both crystalline and amorphous regions, and the arrangement of these two phases directly influences its degradation behavior.

Studies have shown that PLA with higher crystallinity, in both film [73–74] and fiber forms [65], exhibits a higher rate of hydrolytic degradation in phosphate-buffered saline (PBS) at pH 7.4. This is attributed to the higher density of hydrophilic (carboxyl –COOH and hydroxyl –OH) and catalytic (carboxyl –COOH) end groups in the amorphous regions between crystalline domains. These terminal groups reduce chain packing in the amorphous regions between crystallites, making them less densely packed than amorphous polymers and more prone to defects due to crystallization within a confined space [75]. As a result, water diffusion in these amorphous regions increases, and the catalytic effect of the carboxyl groups may accelerate the hydrolytic degradation of semicrystalline samples [20,65,73].

Furthermore, the crystallinity of the encapsulated drug also impacts its release profile. Uncontrolled crystallization of the drug can affect its distribution, leading to the formation of crystals on the fiber surface. In contrast, an amorphous form tends to be more uniformly incorporated into the fiber matrix. Such nonuniform surface distribution may compromise encapsulation efficiency and alter release kinetics [76].

### Surface hydrophilicity

**Figure 10:**
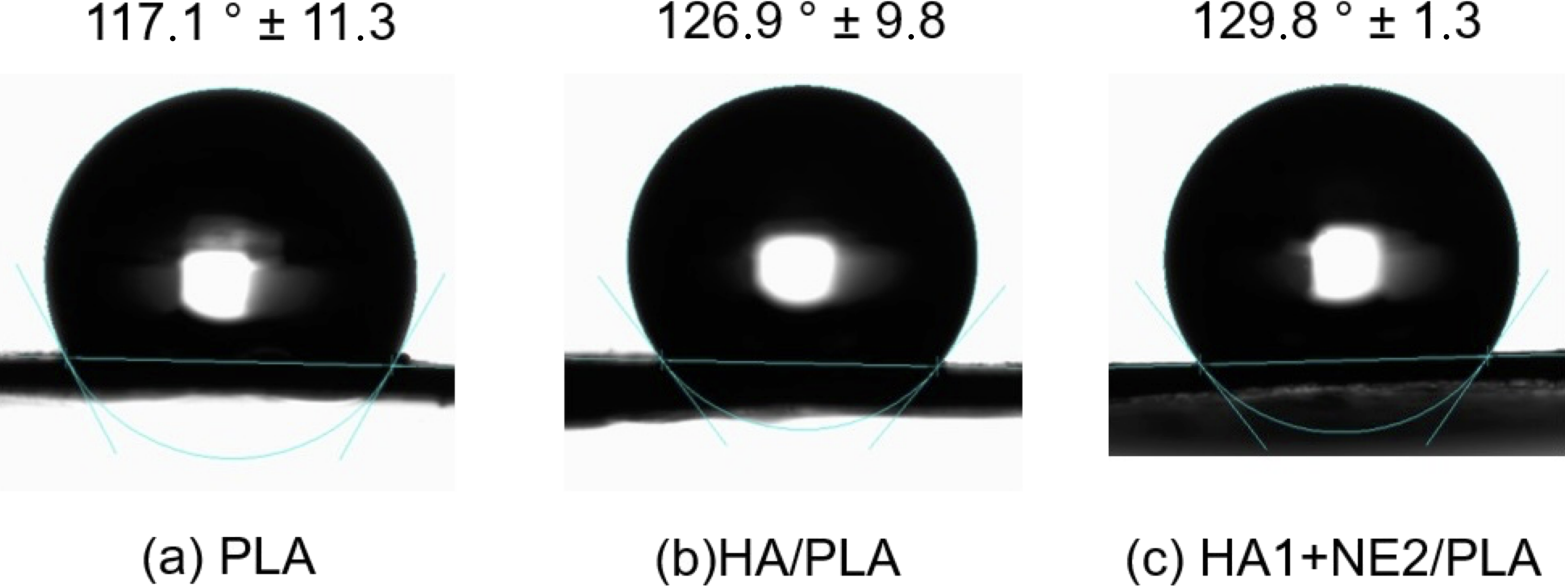
Water contact angles of electrospun nanofibers from the surface of the samples: (A) monolithic nanofiber composed of PLA 20%_(w/w)_, (B) nanofiber composed of HA 1%_(w/w)_ and PLA 20%_(w/w)_, and (C) nanofiber composed of HA 1%_(w/w)_, NE 2%_(w/w)_ and PLA 20%_(w/w)_ electrospun.

The hydrophilicity of the membranes was evaluated through contact angle measurements. Figure 10 shows photographs of a water droplet in contact with the surface of the samples: (a) monolithic PLA, (b) HA/PLA, and (c) HA+NE2/PLA. All samples exhibited a hydrophobic surface, with contact angles ranging between 90° and 150°, attributed to the PLA surface. This result corroborates other characterizations demonstrating that the shell effectively coats the hydrophilic core.

As expected, the monolithic PLA nanofiber sample showed a contact angle of 117.1° ± 11.3°. For core–shell samples, contact angles of 126.9° ± 9.8° and 129.8° ± 1.3° were observed for the samples without NE (HA/PLA) and with NE (HA+NE2/PLA), respectively. The maintenance of surface hydrophobicity with the formation of the core–shell structure indicates the absence of core material at the surface, and that the shell structure does not possess pores that would allow permeation or direct contact of water molecules with the core, as evidenced by scanning electron microscopy (SEM) images.

Electrospun nanofibers organize into an interconnected nanoarchitecture, forming a membrane with porosity that enhances surface hydrophobicity and promotes strong adhesion forces with water [61]. In core–shell nanofibers, the use of PLA as the shell inherently implies that the nanofibers will exhibit a hydrophobic surface due to the high hydrophobicity of this polymer. In cases where not all the hydrophilic core content is fully coated by the shell, a reduction in membrane hydrophobicity is observed due to the presence of hydrophilic nanofibers [77].

Surface hydrophilicity assays revealed that the produced nanofibers exhibit high hydrophobicity, indicating strong anti-bioadhesive properties. This feature is particularly advantageous for preventing membrane adhesion to tissues, such as in tendon and abdominal surgeries [78], and can also reduce bacterial adhesion [79], enhance resistance to moisture, and protect the hydrophilic core from premature degradation. Although such high hydrophobicity may limit direct cell interactions, it can be controllably modulated through surface modifications, such as plasma treatment [80], photopolymerization [81], or chemical grafting [82]. These approaches introduce polar functional groups and tune wettability without altering the internal fiber composition. When combined with the presence of hyaluronic acid in the core, which provides biofunctional potential, these strategies broaden the scope of possible biomedical applications, ranging from wound healing and tissue engineering to the controlled release of bioactive molecules [83–85].

## Conclusion

The results of this study demonstrate the potential of PLA coaxial nanofibers as hybrid systems for the controlled release of bioactive compounds, particularly in topical administration of both hydrophilic and lipophilic agents, such as hyaluronic acid and β-caryophyllene. The application of coaxial electrospinning allowed the formation of well-defined core–shell structures, as confirmed by morphological, spectroscopic, and thermal analyses. Efficient core encapsulation was evidenced by confocal microscopy, FTIR spectroscopy, and selective core removal tests, which indicated the effective isolation of the bioactive components by the PLA shell. From a physicochemical perspective, the fibers presented good thermal stability without compromising the properties of the base polymer. The variation of electrospinning parameters revealed that small changes in core flow rate, HA, and NE-βCp concentration, as well as electrical voltage, directly influence the morphology of the fibers, affecting their uniformity and the presence of structural defects in the form of beads.

Additionally, the presence of porosities in the shell, resulting from the removal of the core, suggests a viable mechanism for the controlled release of active ingredients through diffusion. The high surface hydrophobicity of the nanofibers, confirmed by contact angle measurements, reinforces their potential use in applications that require low tissue adhesion, such as non-adherent dressings. However, for applications where cell adhesion is desired, surface modifications may be necessary. Thus, the data presented here significantly contribute to the understanding of polymer–lipid interactions in coaxial systems and pave the way for the development of multifunctional nanofibers in tissue engineering and controlled drug delivery. Despite these promising results, the present study has some limitations. The encapsulation efficiency and release kinetics of the bioactive compounds were not quantified, and biological assessments were not performed at this stage. Furthermore, morphological instabilities observed at higher core flow rates limited the range of processing conditions which could be explored. These limitations underscore the need for further investigations to establish the translational potential of the proposed nanofiber systems. Future studies will therefore focus on evaluating encapsulation efficiency and conducting in vitro release assays, employing transmission electron microscopy for more detailed visualization of the core–shell structure, and performing biological assessments such as cytocompatibility and scratch wound healing tests to validate the performance of these nanofibers as bioactive dressings. Additionally, surface modification strategies may be explored to tailor adhesion properties according to the intended biomedical application.

## Experimental

### Materials

Tween^®^ 80 (polyoxyethylene (20) sorbitan monooleate), Span^®^ 80 (sorbitan monooleate), β-caryophyllene (purity ≥80%), fluorescein, poly(lactic acid) (*M*_w_ ≈ 93,156 Da), anhydrous chloroform (PA), and PBS tablets (pH 7.2–7.6) were obtained from Sigma-Aldrich (USA). Captex^®^ 300 was kindly provided by ABITEC Corporation (USA). Pharmaceutical-grade hyaluronic acid (95% purity) was purchased from Shandong Focuschem Biotech Co. (China), saline solution (0.9% sodium chloride) from Needs (Brazil), and *N*,*N*-dimethylformamide (DMF, PA) from Vetec Química Fina Ltda (Brazil).

### Manufacturing nanoemulsion via the sonication method

βCp-loaded nanoemulsions were prepared by the sonication method. Briefly, an aqueous and an oil phase were prepared under magnetic stirring, with their composition specified in Table 4. Subsequently, the aqueous phase was carefully added to the oil phase. The mixture was subjected to vortex (IKA) mixing for 5 min, followed by ultrasonication for 5 min at an amplitude of 40% using an ultrasonic device (Eco-sonics/Brazil).

**Table 4:** Composition of the β-caryophyllene-loaded nanoemulsions.

Excipients	% (w/w)

β-caryophyllene	2.5
Captex^®^ 300	2.5
Span^®^ 80	1.4
Tween^®^ 80	3.6
distilled water	90.0

### Electrospinning of nanofibers

#### Coaxial electrospinning setup

The coaxial electrospinning apparatus consisted of an acrylic enclosure to isolate the system, a high-voltage power supply (positioned atop the enclosure) connected to the tip of a core–shell needle, two independent syringe pumps, and a grounded collector. The relative humidity within the enclosure was maintained between 39% and 60% using a dehumidifier and an air conditioning system, ensuring consistent fiber formation conditions.

#### Preparation of polymer solutions

For the shell solution, a 20% (w/v) PLA solution was prepared by dissolving PLA in a 2:8 (v/v) mixture of DMF and chloroform. The core solution was composed of HA at varying concentrations, solubilized in 0.9% (w/v) sodium chloride solution (physiological saline) under constant stirring at room temperature until fully dissolved. To incorporate the nanoemulsion into the fiber core, 2% and 5% (w/w), of NE relative to the total core solution were added to the HA solution and stirred for 1 h to ensure homogeneity.

#### Fabrication of coaxial nanofibers

Coaxial nanofibers were produced using two plastic syringes – one loaded with the PLA shell solution and the other with the HA solution or a mix of HA+NE to the core. Each syringe was connected to an individual syringe pump for independent control of flow rate. The coaxial needle assembly comprised two stainless steel needles concentrically aligned, with an 18G outer needle for the shell solution and a 20G inner needle for the core solution, coaxially fixed.

#### Fabrication of monolithic PLA nanofibers

For comparative purposes, monolithic PLA nanofibers were produced using the same 20% (w/v) PLA solution. An 18-gauge needle connected to a 5 mL plastic syringe was used, operated with a single syringe pump. Electrospinning parameters were as follows: an applied voltage of 17 kV, a flow rate of 0.25 mL/h, a needle-to-collector distance of 12 cm, and a processing time of 5 h.

### Physicochemical characterizations

#### Droplet size

NE-βCp were characterized for hydrodynamic diameter (*z*-average) and PDI using dynamic light scattering (DLS) with a Zetasizer Nano ZS (Malvern Instruments, UK) at 25 °C. The measurements were based on a refractive index of 1.34 for the material. Prior to analysis, the samples were diluted at a ratio of 1:10 (βCp/distilled water). The reported parameters represent the average of readings performed in triplicate for each sample analyzed using the ZetaSizer software 7.11.

#### Scanning electron microscopy

The morphology of the obtained nanofibers (porosity, average diameter, uniformity, and roughness) was evaluated using a scanning electron microscope under different production conditions. This evaluation allowed for the observation of the cross-sectional structure before and after core removal. The nanofibers were coated with approximately 10 nm of gold via sputter deposition for 1 min at 30 mA. Micrographs were acquired using an acceleration voltage of 2 kV and 0.21 nA on a Nova 600 NanoLab system (FEI Company, Thermo Fisher Scientific, USA). The average diameter of the nanofibers (*n* = 120) was calculated using the SizeMeter software. Forty fibers were measured in three distinct regions of the same sample, and the mean value was obtained.

#### Cross-sectional analysis

SEM imaging of the cross-section of the HA+NE2/PLA sample was performed before and after core removal to observe the core–shell structure. For core removal, a portion of the sample was washed multiple times with distilled water, followed by ethanol, and then dried at room temperature. Samples, both before and after washing, were immersed in liquid nitrogen and fractured using tweezers. The fractured samples were mounted on 45 degree stubs to capture cross-sectional images without requiring the sample holder to be moved.

#### Confocal microscopy

Confocal microscopy was conducted to observe the distribution of the core within nanofibers produced via coaxial electrospinning. A disk scanning unit confocal microscope (Olympus, Japan) was utilized for this analysis. Fluorescein was added to the core solution (1% HA + 2% NE) to enable fluorescence imaging during microscopic observation.

#### Thermogravimetric analysis

The thermal stability of the nanofibers was investigated using a Q500 thermogravimetric analyzer (TA Instruments, USA) equipped with platinum (Pt) crucibles. Mass loss as a function of temperature was recorded from 30 to 700 °C at a heating rate of 10 °C/min under a nitrogen atmosphere. Samples analyzed included monolithic PLA nanofibers, HA/PLA, HA+NE2/PLA, and HA powder.

#### Differential scanning calorimetry

DSC analysis was performed using a Q2000 differential scanning calorimeter (TA Instruments, USA) to assess the impact of core–shell formation and NE incorporation on the thermal properties of the nanofibers. Key parameters evaluated included: glass transition temperature (*T*_g_), crystallization temperature upon cooling (*T*_cc_), melting temperature (*T*_m_), melting enthalpy (Δ*H*_m_), and crystallization enthalpy (Δ*H*_cc_). Furthermore, the degree of crystallinity (*X*_c_%) of PLA within the nanofibers was calculated. Samples (monolithic PLA, HA/PLA, and HA+NE2/PLA) were subjected to three heating/cooling cycles. Each cycle involved heating to 200 °C at a rate of 10 °C/min under nitrogen, holding for 1 min, and then cooling at the same rate. The crystallinity of the PLA shell (*X*_c_%) was calculated using the following equation:


[1] $$X_{\text{c}}\%=\frac{\Delta H_{\text{m}}-\Delta H_{\text{cc}}}{X_{\text{PLA}}-\Delta H_{\text{m}0}},$$

where Δ*H*_m0_ is the melting enthalpy of fully crystalline PLA (93.1 J/g), and *X*_PLA_ is the mass fraction of PLA in the fiber (Vidal et al., 2022) [68].

#### Fourier transform infrared spectroscopy

FTIR analysis was conducted to examine the surface composition of the nanofibers and confirm core encapsulation within the PLA shell. An ATR-FTIR spectrometer (Frontier FT-IR/FIR, PerkinElmer, USA) was used to acquire spectra in the range of 4000–600 cm^−1^ with a resolution of 4 cm^−1^ and 60 scans. Samples analyzed included monolithic PLA nanofibers, HA/PLA, HA+NE2/PLA, and HA powder.

#### X-ray diffraction

XRD analyses of PLA nanofibers (monolithic), HA/PLA, HA+NE2/PLA, and HA and PLA powders were performed to investigate and characterize the crystalline structure of the samples and their polymers. Nanofiber samples were cut into circular shapes with a diameter of 2.5 cm and placed on the sample holder. For polymer powders, sieving was conducted using an ASTM 70 mesh (212 µm) before compressing the powders onto the sample holder to ensure grain homogeneity. The analyses were carried out using a LabX XRD-6100 diffractometer (Shimadzu, Japan) in an angle ranging from 2° to 50° (2θ), using a step size of 0.02° (2θ), and Cu Kα radiation (λ = 1.5418 Å) with a voltage of 40 kV and a current of 30 mA. The degree of crystallinity (*X*_c_%) of each sample was calculated from the diffractogram using Equation 2 (Madsen et al., 2011) [72]. The areas of the peaks in the diffractograms were determined using OriginPro 8.5 software (OriginLab Corporation).


[2]

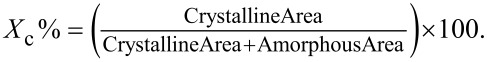



#### Contact angle analysis

Contact angle measurements were conducted to evaluate the surface wettability of PLA nanofiber samples, including monolithic PLA, HA/PLA, and HA+NE2/PLA, using an OCA 15EC optical contact angle tensiometer (Dataphysics Instruments, Germany). Samples were cut into 1.5 cm × 2 cm sections and fixed to a metal holder. Afterward, a droplet of distilled water was placed on three distinct regions on the surface of each sample, and images were captured to determine the contact angle. The final contact angle value of each sample was calculated as the average of the three measurements.

#### Statistical analysis

All samples were produced in triplicate. Statistical analysis was performed using OriginPro 8.5 software (OriginLab Corporation). The diameters of the obtained nanofibers were quantified from the micrographs using SizeMeter.

## Data Availability

Data generated and analyzed during this study is available from the corresponding author upon reasonable request.
